# Dynamics of glacier calving at the ungrounded margin of Helheim Glacier, southeast Greenland

**DOI:** 10.1002/2015JF003531

**Published:** 2015-06-01

**Authors:** Tavi Murray, Nick Selmes, Timothy D. James, Stuart Edwards, Ian Martin, Timothy O'Farrell, Robin Aspey, Ian Rutt, Meredith Nettles, Tim Baugé

**Affiliations:** ^1^ Glaciology Group, Department of Geography, College of ScienceSwansea UniversitySwanseaUK; ^2^ School of Civil Engineering and GeosciencesNewcastle UniversityNewcastleUK; ^3^ Department of Electronic and Electrical EngineeringUniversity of SheffieldSheffieldUK; ^4^ Lamont‐Doherty Earth ObservatoryColumbia UniversityNew YorkNew YorkUSA; ^5^ Thales, Research & TechnologyBerkshireUK

**Keywords:** Glacier calving, Iceberg calving, Greenland

## Abstract

During summer 2013 we installed a network of 19 GPS nodes at the ungrounded margin of Helheim Glacier in southeast Greenland together with three cameras to study iceberg calving mechanisms. The network collected data at rates up to every 7 s and was designed to be robust to loss of nodes as the glacier calved. Data collection covered 55 days, and many nodes survived in locations right at the glacier front to the time of iceberg calving. The observations included a number of significant calving events, and as a consequence the glacier retreated ~1.5 km. The data provide real‐time, high‐frequency observations in unprecedented proximity to the calving front. The glacier calved by a process of buoyancy‐force‐induced crevassing in which the ice downglacier of flexion zones rotates upward because it is out of buoyant equilibrium. Calving then occurs back to the flexion zone. This calving process provides a compelling and complete explanation for the data. Tracking of oblique camera images allows identification and characterisation of the flexion zones and their propagation downglacier. Interpretation of the GPS data and camera data in combination allows us to place constraints on the height of the basal cavity that forms beneath the rotating ice downglacier of the flexion zone before calving. The flexion zones are probably formed by the exploitation of basal crevasses, and theoretical considerations suggest that their propagation is strongly enhanced when the glacier base is deeper than buoyant equilibrium. Thus, this calving mechanism is likely to dominate whenever such geometry occurs and is of increasing importance in Greenland.

## Key Points

- GPS sensor network and oblique cameras installed at Helheim Glacier during 2013
- Calving by bottom‐crevassing with ice downglacier of flexion zones rotating
- Mechanism likely to dominate whenever glacier is out of buoyant equilibrium

## 1 Introduction

Iceberg calving is an important mass loss mechanism for ice sheets and tidewater glaciers. The Greenland Ice Sheet is losing mass at an accelerating rate [*Velicogna*, 2009] due to both increased runoff and increased discharge of icebergs. During the late 1990s and the first decade of the 21st century, many of Greenland's tidewater glaciers accelerated, thinned, and retreated [*Rignot and Kanagaratnam*, 2006]. The resulting dynamic thinning penetrates deep into the ice sheet and dominates mass loss from southeast Greenland and other regions of the ice sheet [*Pritchard et al*., 2009]. Overall, the ice sheet's thinning rate is significantly higher at the calving margins of marine‐terminating glaciers than at those that are land terminating [*Sole et al*., 2008]. Knowledge of calving processes and rates are fundamental to understanding the dynamics of marine‐terminating glaciers worldwide and to predictions of the future contribution of the Greenland and Antarctic Ice Sheets to global sea level.

Glacier calving was originally modeled using empirical relationships that link the calving rate to water depth [*Pelto and Warren*, 1991]. It was subsequently suggested that a glacier calves when the height of its calving cliff above flotation reaches a critical threshold [*van der Veen*, 1996]. However, neither of these empirical models can describe the full range of behavior at tidewater glaciers, which can, for example, form floating tongues. More recent models include the crevasse depth model initially proposed by *Benn et al*. [2007], which predicts that calving occurs when surface crevasses propagate to sea level. *Krug et al*. [2014] proposed a more sophisticated combined continuum damage mechanics and fracture mechanics model that still focused on surface crevassing. However, if the ice is floating or ungrounded, both observations [*Warren et al*., 2001; *Boyce et al*., 2007; *James et al*., 2014] and models [*Nick et al*., 2010; *Bassis and Jacobs*, 2013] suggest that buoyancy forces and the formation and propagation of basal crevasses also become important. Basal crevasses are expected to form on floating ice or in locations close to the grounding line where basal water pressure and ice extension rates are high [*van der Veen*, 1998], so we would expect them to form close to the margins of many of Greenland's tidewater glaciers where these conditions occur.

**Figure 1:**
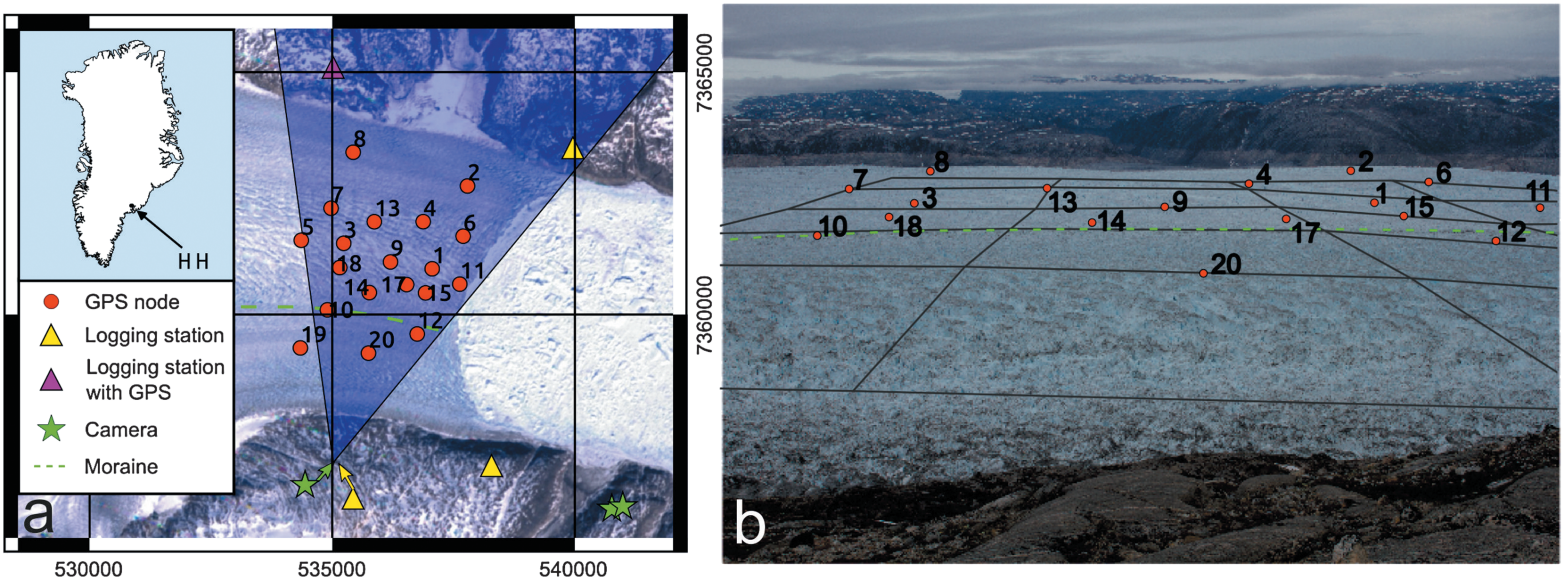
Location of instruments at Helheim Glacier close to the start of the field campaign. The instrument positions are plotted for DOY 198, 2 days after installation, since node 18 did not start to transmit valid data until the end of DOY 197. Location of moraine referred to in text shown by dashed green line. This moraine can also be seen (without annotation) in Figure 6. (a) Vertical view, shaded area shows field of view for side‐viewing camera used for tracking. Background image is Landsat 7 image from 16 June 2013 with SLC lines removed graphically for visual continuity, image chosen with margin closest to that at date of instrument installation. Grid is UTM zone 24 in meters. Due to the close proximity of the SW logging station and the side‐looking cameras, positions are indicated with arrows for clarity. Insert shows location of glacier in Greenland (HH). (b) Nodes superimposed on image from side‐viewing camera 4; two nodes (5 and 19) are at this time out of the camera view to the left (west). One kilometer grid superimposed to aid image interpretation. DEM of glacier surface used for grid was created by combination of lidar flown on DOY 197, filled where needed with DOY 198 photogrammetric DEM and lidar flown on DOY 222.

Helheim Glacier (Figure 1) is one of the largest tidewater glaciers in Greenland, draining some 52,000 km^2^ of the Greenland Ice Sheet and terminating in Helheim Fjord, an arm of Sermilik Fjord. The glacier and its fjord systems have been intensely studied in part because of its accessibility [e.g., *Nettles et al*., 2008; *de Juan et al*., 2010; *Straneo et al*., 2011; *Sutherland and Straneo*, 2012; *Jackson et al*., 2014]. The glacier's calving margin is around 6 km wide, and ice at the margin currently flows at rates of around 20–25 m/d [*Bevan et al*., 2012]. In the early 2000s, the glacier accelerated, thinned, and retreated [*Howat et al*., 2005; *Luckman et al*., 2006; *Stearns and Hamilton*, 2007], and during 2005 it was at its most retreated position. The glacier subsequently readvanced in 2006, slowed [*Howat et al*., 2007], and ceased thinning [*Stearns and Hamilton*, 2007; *Howat et al*., 2007], but maintained a faster flow rate than prior to its retreat [*Murray et al*., 2010].

Warm water of subtropical origin has been observed in Sermilik and Helheim fjords at depths greater than ~250 m [*Straneo et al*., 2010, 2011], and in other regions warm water has been shown to cause undercutting of the glacier front and enhanced calving rates [e.g., *Motyka et al*., 2003]. Melt rates due to this heat source at the front of Helheim Glacier are estimated to be ~1.8 m/d [*Sutherland and Straneo*, 2012]. Models however do not currently agree whether undercutting at the glacier plays a major role in calving [*Nick et al*., 2010, 2013; *O*'*Leary and Christoffersen*, 2013; *Cook et al*., 2014].

*Nettles et al*. [2008] reported on the results of a network of 11 GPS nodes installed at Helheim Glacier for ~50 days during 2007. Calving occurred coincidentally with the occurrence of glacial earthquakes [*Joughin et al*., 2008; *Nettles et al*., 2008] and resulted in step increases in the flow speed of the glacier, with the greatest increase at the calving margin [*Nettles et al*., 2008]. Tidally driven variation in horizontal velocity was reported, but no tidally driven changes in vertical elevation of the glacier surface, suggesting that the glacier front (or at least the portions of the glacier that were instrumented) was grounded during 2007 and 2008 [*de Juan et al*., 2010]. Observations at Jakobshavn Isbræ, west Greenland, [*Rosenau et al*., 2013] show increases of flow speed during calving as well as vertical displacements prior to calving that are comparable with observations at Helheim Glacier.

In contrast, during 2010 and 2011, observations of Helheim Glacier from daily digital elevation models derived from oblique stereo terrestrial photogrammetry were interpreted to show that calving occurred driven by buoyant flexure interpreted as resulting from the propagation of basal crevasses [*James et al*., 2014]. Four major calving events in 2010 and one in 2011 were studied. However, in neither year was it possible to state categorically that the glacier front was ungrounded at the time of calving, although this is essential for buoyant flexure to occur.

Calving at Helheim Glacier occurs year round, and there is a relatively weak seasonal cycle of frontal position at the glacier compared to its neighbor, Kangerdlugssuaq Glacier [*Bevan et al*., 2012]. Few tabular icebergs are calved at the glacier under current conditions and most calving events result in icebergs that overturn with the top surface toppling inward toward the calving front (i.e., bottom out), but a small number of outward toppling events also occur [*James et al*., 2014].

In this paper we report the detailed dynamics of the margin of Helheim Glacier during a series of major calving events during summer 2013 recorded using a wireless network of custom‐built GPS receive‐transmit nodes. The previous projects discussed above have reported the calving process using daily averaged flow rates with more limited higher‐rate observations [*Nettles et al*., 2008] or daily measurements of surface elevation and displacement [*James et al*., 2014]. The key differences between the results we report and those from previous studies are our high data rate, the proximity of our nodes to the calving front, and the real‐time communication of GPS data from the glacier. These differences mean that GPS position measurements are available right to the moment of node loss at the time of iceberg calving and we can demonstrate unequivocally that the glacier front is ungrounded during the observed calving events. The high temporal sampling of the GPS data interpreted in combination with daily tracking on oblique camera imagery, which give excellent spatial data coverage, provides a uniquely rich record of calving at the glacier margin.

## 2 Methodology

We designed and operated a wireless network of GPS nodes at the margin of Helheim Glacier (Figure 1). The GPS data received by the nodes was then transmitted every few seconds to logging stations positioned on bedrock adjacent to the glacier. Field trials with a small network in 2012 were followed by a full 20‐node network during 2013. In 2013 nodes were installed between day of year (DOY) 192 and 196 (11–15 July) and the site was visited again to retrieve surviving nodes on DOY 240 (28 August). Five cameras were also installed on the bedrock. These cameras captured the glacier front hourly in order to provide information on front location and to allow the production of digital elevation models (DEMs) using the cameras as stereo pairs and feature tracking between images [*James et al*., 2014]. We used data from the Global Seismographic Network (GSN) to determine the timing of glacial earthquakes located at Helheim Glacier [*Ekström et al*., 2006; *Nettles and Ekström*, 2010]. We also inspected GSN array stacks interactively to identify smaller glacial earthquakes not detected by the standard algorithm. Initial earthquake detection was conducted independently of the image analysis. We used the cameras and seismograms together to determine the precise timing and size of iceberg calved. We define the size of the icebergs by the amount of ice lost from the glacier although the icebergs typically break into smaller pieces as they rotate.

### 2.1 GPS Network

The 2013 GPS network consisted of 19 on‐ice GPS receiver nodes (20 were installed, but one (node 16) never communicated) and four logging stations installed on bedrock, one of which (NW station) was co‐located with a GPS base station. At the end of the 2013 deployment five ice nodes were collected having run through the entirety of the field deployment. Zigbee transceivers were used to transmit the GPS data from the ice nodes to the logging stations [*Baronti et al*., 2007]. Zigbee transceivers are designed for hostile radio frequency (RF) environments and provided a low‐power, low‐cost wireless network with automatic data packet retransmissions and automatic network formation. The logging stations also acted as Zigbee network coordinators controlling data collection from the ice nodes. Field trials during 2012 showed it was common for ice nodes to lose line of sight to a single logging station behind ice pinnacles as they moved downglacier [*Martin et al*., 2014]. Therefore, in 2013, four logging stations were used, two on each side of the glacier, to record ice node data. Each ice node then transmitted independently to two logging stations which divided the network into four 5‐node subnetworks. Zigbee uses carrier sense multiple access to avoid transmission collisions; however, on the glacier this functionality is severely inhibited as the highly crevassed surface shields the ice nodes from one another. Data packet collisions within a subnetwork were largely avoided by employing a logging station round‐robin scheme, which, within each subnetwork, polled each node in turn with a message to which the nodes replied with GPS data.

The ice nodes were purpose built and contained a dual‐frequency GPS board attached to a geodetic‐grade antenna and two Zigbee communications boards coupled to a single wireless antenna using an RF splitter circuit. Each Zigbee board communicated separately with a logging station, providing redundancy in data transmission. Each ice node was powered by a battery and solar panel. The network nodes were emplaced by helicopter and secured with crampons (15 nodes) or crampons and stakes drilled into the ice (five nodes). Known causes of node communication loss were due to calving or because the node tipped over orienting the antennas unfavourably.

The GPS base station employed a dual frequency GPS board linked to a geodetic‐grade antenna located some 20 m from the receiver unit and installed on a permanently established, highly stable geodetic monument. The coordinates of the base station were calculated using GIPSY OASIS II software v6.2 from the NASA Jet Propulsion Laboratory (JPL), and daily static coordinates were estimated using the software in Precise Point Positioning (PPP) mode [*Zumberge et al*., 1997]. JPL‐derived GPS satellite products were used to enable ambiguity resolution for final coordinate estimation [*Bertiger et al*., 2010]. Ocean tide loading was modeled using the FES 2004 model [*Lyard et al*., 2006]. In addition to the base station coordinates and receiver clock offset, a zenith wet tropospheric delay and tropospheric gradients were estimated as a random walk process at 300 s intervals [*Bar‐Sever et al*., 1998]. The Niell mapping function [*Niell*, 2000] was used to map the tropospheric delay with a minimum elevation cutoff of 10°. A linear velocity model was fitted to the observation period to account for bedrock uplift [*Bevan et al*., 2015] with repeatabilities of <2 mm in plan and 4 mm in vertical.

Processing of the ice node GPS data was performed using the Track 1.29 module of the GAMIT‐GLOBK10.50 GPS processing software [*Chen*, 1998]. Track uses the dual frequency carrier phase relative positioning method and requires GPS observations from a reference station at known coordinates. All ice nodes were positioned relative to the GPS base station (Figure 1). Due to the distance of the nodes from the base station (3–8 km), the ionosphere free linear combination (LC) of L1 and L2 carrier phase GPS observations was used in addition to satellite orbit and high‐rate clock products tabulated at 5 s intervals from CODE [*Dach et al*., 2009]. The clock products are aligned to the ionosphere free P1 and P2 code phase combination and so differential code biases (DCB) from Astronomisches Institut Universität Bern AIUB were applied to “correct” C1 (Course/Acquisition code) to P1. IGS08 absolute antenna phase center offset and variation corrections were applied.

On‐ice node positions were estimated for each reported GPS observation. Positions were modeled as random walk stochastic process with process noise of 0.01 m/s^0.5^. While this is a considerably looser constraint than is needed to model the ~0.3 mm/s motion of the glacier, we wanted to avoid overconstraining any rapid motion during calving events. Due to the relatively short baseline lengths, ~1–7 km, no tropospheric correction was estimated as at times the low number of visible satellites degraded the final solution if this parameter estimation was included in the solution. GPS data were then filtered by excluding from plots any data where the number of unfixed biases was greater than 2, the number of double differences was less than 4, or the height uncertainty was greater than 0.1 m. Precision of the resulting coordinates based on Track formal errors and assessment of the detrended time series outside of calving events for are ~2 cm in north, 1 cm in east, and 2.5 cm in height.

### 2.2 Camera Methodology

Cameras were installed in stereo configuration at two locations on the south side of the fjord (Figure 1). An 18 megapixel Canon 7D digital Single Lens Reflex (dSLR) camera was located close to the SW base station and viewed the glacier orthogonally across its frontal part (essentially sideways looking). Two 15.1 megapixel Canon 50D dSLR cameras were located viewing the glacier calving front obliquely (essentially front looking). The cameras had fixed 28 mm focal length lenses and were powered using solar charged 12 V batteries. External timers were manually synchronized and set to take hourly photographs between DOY 194 or 196 and DOY 245 (13 July or 15 July to 2 September). Timer drift was found to be < 5 s over the observation period. The side‐looking camera was used for tracking to detect glacier motion between images (described below), and the front‐looking cameras were used to produce DEMs of the glacier which were used to map calving front positions. The supplementary material to *James et al*. [2014] details the methodology used to produce DEMs.

GPS nodes were plotted on each photograph using the collinearity condition of photogrammetry which requires that a point on the ground, the camera center, and the corresponding point on the image plane lie in a straight line [*Wolf and Dewitt*, 2000]. This relationship, subject to errors of lens distortion, provides the *x‐y* image coordinates of each node using (i) its XYZ position in ground space; (ii) the camera calibration parameters, focal length, and principle point; and (iii) the exterior orientation parameters of the camera including its XYZ position in ground space and azimuth, tilt, and swing orientation angles.

Tracking was undertaken on images from the sideways‐looking camera to detect motion in the vertical and horizontal image directions analogous to that reported in *James et al*. [2014] but using a different algorithm. The OpenCV‐Python implementation of the dense optical flow method was used [*Farneback*, 2003] with search window of 16 pixels, size of the pixel neighborhood to find polynomial expansion in each pixel set to 5, and seven iterations. The standard deviation of the Gaussian used to smooth derivatives as the basis for the polynomial expansion was 1.1. Five pyramid layers were used with each layer half the size of the previous one.

Tracking follows recognizable patterns (typically crevasses) from image to image as the glacier flows. Images with different lighting or obstruction (such as fog and changes in cloud cover causing shadows on the ice surface or changes in sun angle) do not track well. We therefore tracked images over 24 h when lighting is most likely to be similar. All daylight images were tracked, and then the best result during each 24 h period was chosen. Systematic tracking errors will result from any camera movement but also from changes in atmospheric conditions and temperature‐dependent lens geometry, both of which cause changes to the path of light entering the camera. For securely mounted cameras, movement is expected to be negligible except during storm events. Since storms were not a problem during our tracking periods, we attribute the majority of systematic errors in our tracking to changes in atmospheric conditions and lens geometry. This systematic bias was assessed and removed by measuring mean displacement in a 700 × 430 pixel window on stable bedrock in the foreground of each image pair and subtracting this value from the tracking results. Of the image pairs shown in this paper, only two required corrections higher than 2 pixels in *x* and 1 pixel in *y*. After these errors were removed, the mean apparent displacement of the bedrock in the mountains in the background of the images (Figure 1b) was 0.033 pixels in the horizontal direction and 0.020 pixels in the vertical direction.

On the sideways‐looking images movement on the *x* axis of the images approximates to downglacier flow. Image movement on the *y* axis of the images is more difficult to interpret as while most sensitive to changes in vertical and crossglacier position of the glacier, it is also sensitive to downglacier flow if the camera is slightly oblique to this flow direction or if the glacier surface is not flat. Crossglacier flow is negligible on these images as typically glacier flow measured on each GPS node varied in direction by only 1–2° over the measurement period. Vertical changes can be dynamic or melt related. Previous measurements have shown that summer ablation rates were ~3.2 cm/d at 650 m elevation [*Andersen et al*., 2010]. The spatial pattern in ablation should be governed by elevation and shading. Clearly, the spatial distribution in *y* axis image movement revealed by our tracking cannot be explained by differences in ablation.

Two aspects of the images should be noted—both related to the relatively low viewing angle of the camera from the glacier side (see camera view on Figure 1a). First, the north side of the glacier appears greatly foreshortened compared to the south side, and the region of the glacier instrumented by the network (largely north of the moraine seen on Figure 1b) occupies a relatively small portion of the camera image (Figure 1). Second, this foreshortening means that tracking is much more sensitive to changes in the close part of the image (south side of the glacier) than those at a distance (north side of the glacier) so a 1 pixel displacement in vertical or horizontal at a distance results from a much larger actual displacement than the same pixel displacement close to the camera. To aid interpretation of the images, Figure 1b displays the glacier surface with a 1 km grid overlain.

Calving events were identified by visual inspection of camera images. Calving events are most easily identified by the large movement of the mélange floating on the fjord that accompanies them, but changes in the calving front itself can also be identified. At the start of the season, the front‐looking images had to be used because the glacier margin initially advanced beyond the field of view of the sideways‐looking cameras. After a major calving event on DOY 205, the sideways‐looking cameras were used as the front remained in view and it is easier to see calving events on these images. Once events were identified by the loss of ice and movement of the mélange between the hourly images, the timing of any glacial earthquake/s detected in this hour was assumed to be the exact time of iceberg calving [*Nettles et al*., 2008].

## 3 Results

Our network was installed over the period DOY 192–196. Once our network was installed, major calving events occurred on DOY 205–206 (a series of more than four events), DOY 211–212 (a series of three events), DOY 220 and DOY 226.

### 3.1 Calving Events and Glacial Earthquakes

Table 1 lists the occurrence of calving events on camera images and glacial earthquakes. The majority of events are seen as both glacial earthquakes and calving events on the camera images. There were 10 events seen on both camera images and as glacial earthquakes; two of these events consisted of two earthquakes in the same hour. There was also one earthquake without a corresponding calving event and one calving event with no corresponding earthquake. All glacial earthquakes detected by the standard algorithm correspond to calving events. The glacial earthquake detected by inspecting GSN stacks with no corresponding synchronous calving event visible in the camera imagery occurred on DOY 205, within a few hours of major calving events, and is a weak seismic signal. A calving event with no corresponding glacial earthquake was seen on DOY 215.

**Table 1:** Calving Events Seen in Camera and Glacial Earthquake Records^a^

DOY 2013	Camera Image Before Event	Camera Image After Event	Size of Event / Side of Glacier	Glacial Earthquake
174	Prior to installation	Prior to installation		14:35
**194/196**	**20:00**	**Start of GPS sequence**		
205				11:01_E_
	*19:00*	*20:00*	*Medium N*	*19:37_E_*
	*19:00*	*20:00*	*Medium N*	*19:38_E_*
*206*	*03:00*	*04:00*	*Large*	*03:13 _E_*
	*12:00*	*13:00*	*Very large N and S*	*12:56*
	*13:00*	*14:00*	*Medium S*	*13:03_E_*
*207*	*01:00*	*02:00*	*Medium S*	*01.43_E_*
*211*	*19:00*	*20:00*	*Very large N and S*	*20:01*
*212*	*19:00*	*20:00*	*Large N and S*	*19:21*
	*20:00*	*21:00*	*Off camera S*	*20:28_E_*
215	00:00/01:00	02:00	?size S?	
*220*	*06:00*	*07:00*	*Large S*	*06:49*
*226*	*23:00*	*227 00:00*	*Large S and central*	*23:41*
	*23:00*	*227 00:00*	*Large S and central*	*23:50*
**245**	**15:00**	**End of GPS sequence**		

a Off camera event was detected by movement of the ice mélange only, during all other events the loss of ice could be seen on the glacier front together with movement of the mélange. Small calving events are not indicated. Data in italics show events that agree between the camera and glacial earthquake records. Size of calving events is from visual inspection of images and is qualitative. N and S refer to the side of the glacier affected (north or south). Glacial earthquakes without subscripts were detected using the standard algorithm; those with subscript E were taken by manual interpretation of GSN stacks.

### 3.2 GPS Network

**Figure 2:**
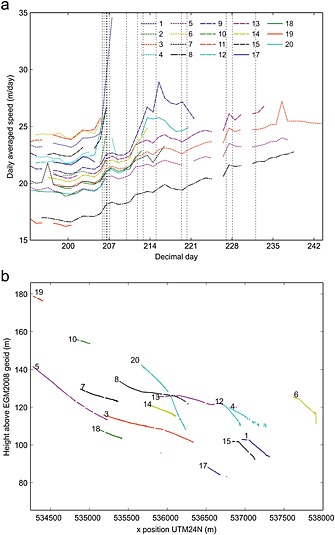
Summary diagram showing results from GPS nodes. For initial locations see Figure 1. (a) Daily velocity. Vertical dashed lines are calving events / glacial earthquakes (Table 1). (b) Vertical elevation of nodes against distance and differences in elevation at the same distance are largely the result of crossglacier topography.

**Figure 3:**
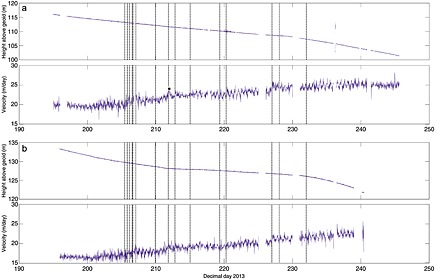
Elevation and downglacier velocity (calculated as best fit over each 15 min data section) for nodes (a) 3 and (b) 8, both of which lasted through almost the entire field season and were collected at the end. For initial locations see Figure 1. Vertical dashed lines are calving events / glacial earthquakes (Table 1). Vertical height is relative to EGM2008 geoid.

**Figure 4:**
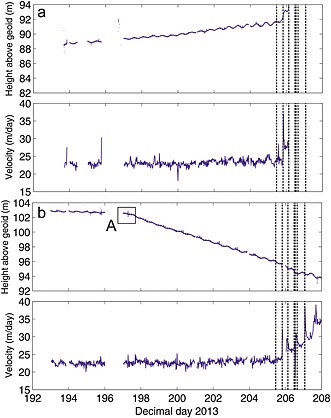
Elevation and downglacier velocity (calculated as best fit over each 15 min data section) for nodes (a) 11 and (b) 1. For initial locations see Figure 1. Node 11 was lost at a calving event on DOY 206 (the last data received from the node was at 03:11:50, 106 s before the corresponding glacial earthquake centroid (Table 1)). Node 1 was lost on DOY 208. The inflexion in the rate of change of vertical position “A” is discussed in the text. Vertical dashed lines are calving events / glacial earthquakes (Table 1). Vertical height is relative to EGM2008 geoid.

**Figure 5:**
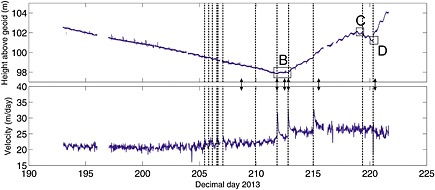
Elevation and downglacier velocity (calculated as best fit over each 15 min data section) for node 9. For initial location see Figure 1. Arrows mark times of images in Figure 6. The last data from node 9 was received on DOY 221 when it was very close to the front. The inflexions in the rate of change of vertical position “B,” “C,” and “D” are discussed in the text. Vertical dashed lines are calving events / glacial earthquakes (Table 1). Vertical height is relative to EGM2008 geoid.

Figure 1 shows the layout of the on‐ice network just after installation. Most nodes increase their daily horizontal flow speed as they advect downglacier (Figure 2a), and their vertical height has a downward trend because they flow downslope and because the glacier surface melts (Figure 2b). Nodes centrally placed on the glacier typically show tidal modulation of flow speed and vertical position in at least some part of their record (e.g., Figures 3, 4, 5) provided they transmit data within a distance ~1.5 km from the glacier margin or closer. Those nodes placed close to the sides of the glacier (e.g., nodes 2, 12, and 20) do not show clear vertical modulation of their position. We interpret vertical modulation of position at tidal frequencies to show that the glacier is ungrounded at that location, suggesting that the sides of the glacier, especially the south side, are probably grounded throughout the field season but that the central part of the glacier is ungrounded.

Nodes 3 and 8 were installed, respectively, 2.8 and 3.6 km from the glacier front (Figure 1). The nodes ran through the majority of the field season and exhibited similar signals. Their overall horizontal flow speed increased through time and showed tidal modulation in flow rate (Figure 3). Tidal modulation of the vertical position of node 3 also becomes apparent toward the end of the record, although increases in tidal amplitude do not seem necessarily associated with the timing of calving events.

Figures 4 and 5 show the flow speed and vertical position of four nodes installed along an approximate flowline. Of these only node 13 was retrieved, whereas all three of the other nodes were located on the part of the glacier that was calved as icebergs during our field season. Node 11 (Figure 4a) was the furthest downglacier of our network and was installed approximately 0.4 km from the glacier front (Figure 1). From installation the node displayed tidal modulation in elevation, suggesting this area of the glacier was not grounded. The amplitude of the modulation increased during the 12 day period (from ~0.2 m to ~0.5 m) until the node was lost during the calving event of DOY 206. The node's overall elevation increased (it experienced uplift) until it was lost, and data were retrieved from the node as it fell or slid off the glacier. There was a strong increase in flow speed which seemed to occur concurrently with the calving event at 19:37/19:38 on DOY 205 (Table 1). Node 1 (Figure 4b) was installed 1.0 km from the glacier front (Figure 1). Like node 11 it showed tidal modulation in its vertical position throughout the 16 day period until it was lost. The node flowed downslope throughout the record, and there was an inflexion in the downslope flow on DOY 197.6 (“A” on Figure 4b). The flow speed of the node increased coincident with calving events on 205–207 and the node was lost on DOY 208.

Node 9 was installed approximately 1.9 km from the glacier front (Figure 1). The node showed weak modulation in its vertical position from installation (~0.05 m in amplitude day 203–205) although this became weaker in the period DOY 210–215 before becoming very clear (~0.2 m in amplitude) after the calving event on DOY 215 (Figure 5a). There was tidal modulation of horizontal flow speed throughout the node record. The vertical position of the node has clear inflexion points (marked as “B,” “ C,” and “D” in Figure 5a). Inflexions “B” and “D” correspond to calving events, inflexion “C” does not. The node's overall elevation decreased until calving events on DOY 211 and 212 (“B”). After the second event the elevation started to increase and the node moved upward until the end of DOY 218 (“C”). It then moved downward for ~18 h before starting to move up again 1 day after the calving event on DOY 220. The node continued to transmit data until DOY 221. The node's flow speed increased coincident with the suite of calving events on DOY 205–206, resulting in a step change from ~20.5 m/d to ~22 m/d. The node has a sharp velocity increase in response to events on DOY 211, 212, and 215; its increase in flow rate in response to the 215 event was from ~25 to ~33 m/d. These sharp increases occurred more rapidly than the corresponding decreases, which in all three cases level out ~0.5 day later at a higher velocity than prior to the event.

**Figure 6:**
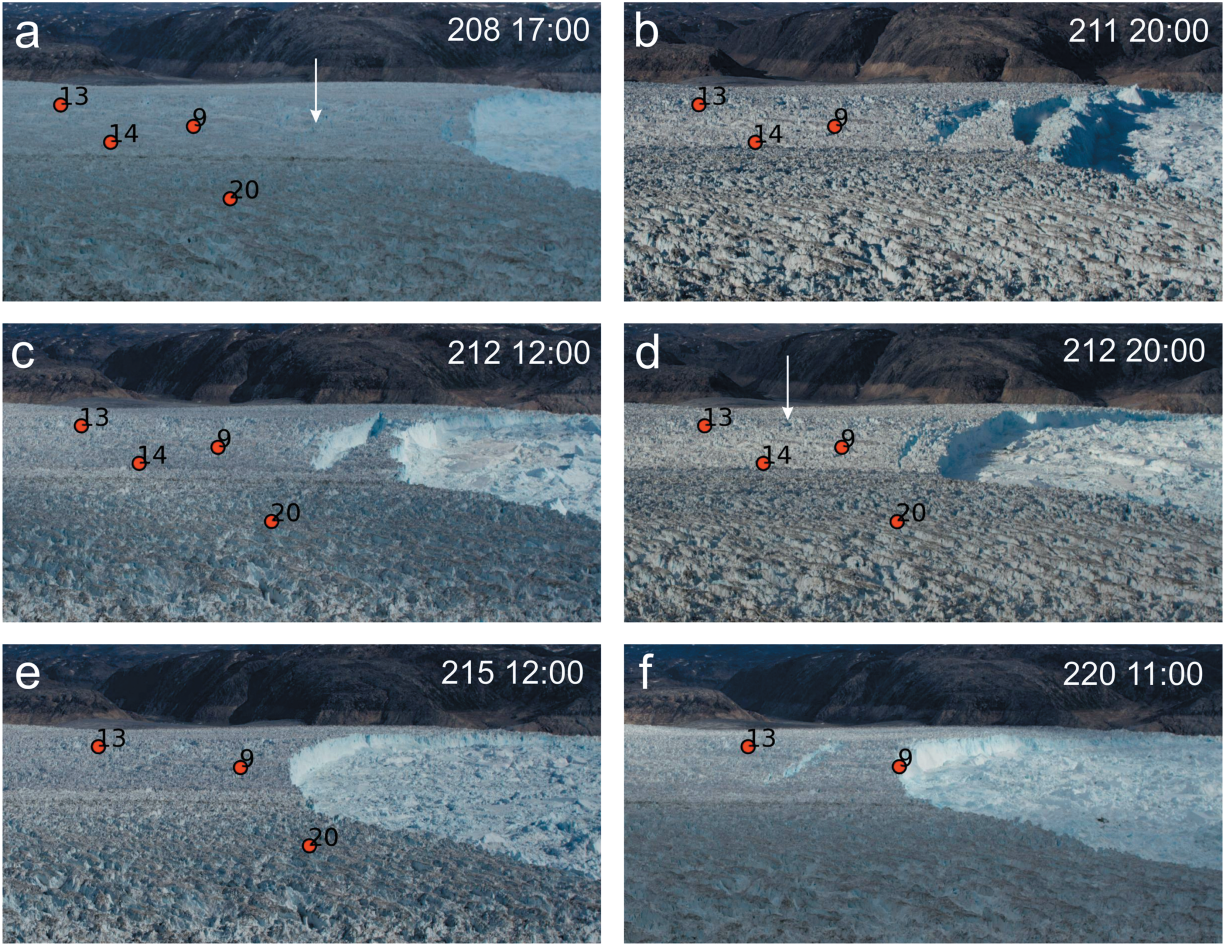
Enlargements of photographs of the glacier front at selected intervals. These times are marked on Figure 5, and the images are selected and described with respect to the behavior of node 9. (a) DOY 208 17:00. The flexion zone can be made out on the glacier marked by arrow. Downglacier of this zone rotation and uplift of the ice was occurring. Node 9, upglacier of the rift, was moving downward. (b) DOY 211 20:00. Node 9 was still moving downward. Flexion zone was very clear, and the glacier was caught midcalving event with the front block rotating. (c) DOY 212 12:00. Once this iceberg had calved node 9 started uplifting slightly (moving upward). (d) DOY 212 20:00. Node 9 was now located downglacier of a newly formed flexion zone (marked by arrow) and therefore on the rotating block and was uplifting. (e) DOY 215 12:00. Vertical uplift was strongly positive. (f) DOY 220 11:00. Node was located very close to margin. Rift / crevasse marking flexion zone was very large.

Figure 6 shows camera images chosen to investigate the inflexions in the vertical position of node 9 (timings shown by arrows on Figure 5). On DOY 208 node 9 was located upglacier of a rift that can clearly be identified on the glacier surface (Figure 6a) at which the glacier later calved. At this time the vertical movement of the node was downward (Figure 5). Images on DOY 211 caught the glacier mid calving event (Figure 6b), when the node immediately sped up and started uplifting (Figure 5), although the rate was low until after events on DOY 212 (Figures 5 and 6c; Table 1). By DOY 215 a new rift was very clear upglacier of node 9 (Figure 6e) and the node uplifted strongly (Figure 5), although there was possibly some surface indication of the rift earlier than this (Figures 6c and 6d). By DOY 220 node 9 was again uplifting strongly (Figure 5) and was located right on the calving margin of the glacier (Figure 6f).

### 3.3 Tracking on Camera Images

**Figure 7:**
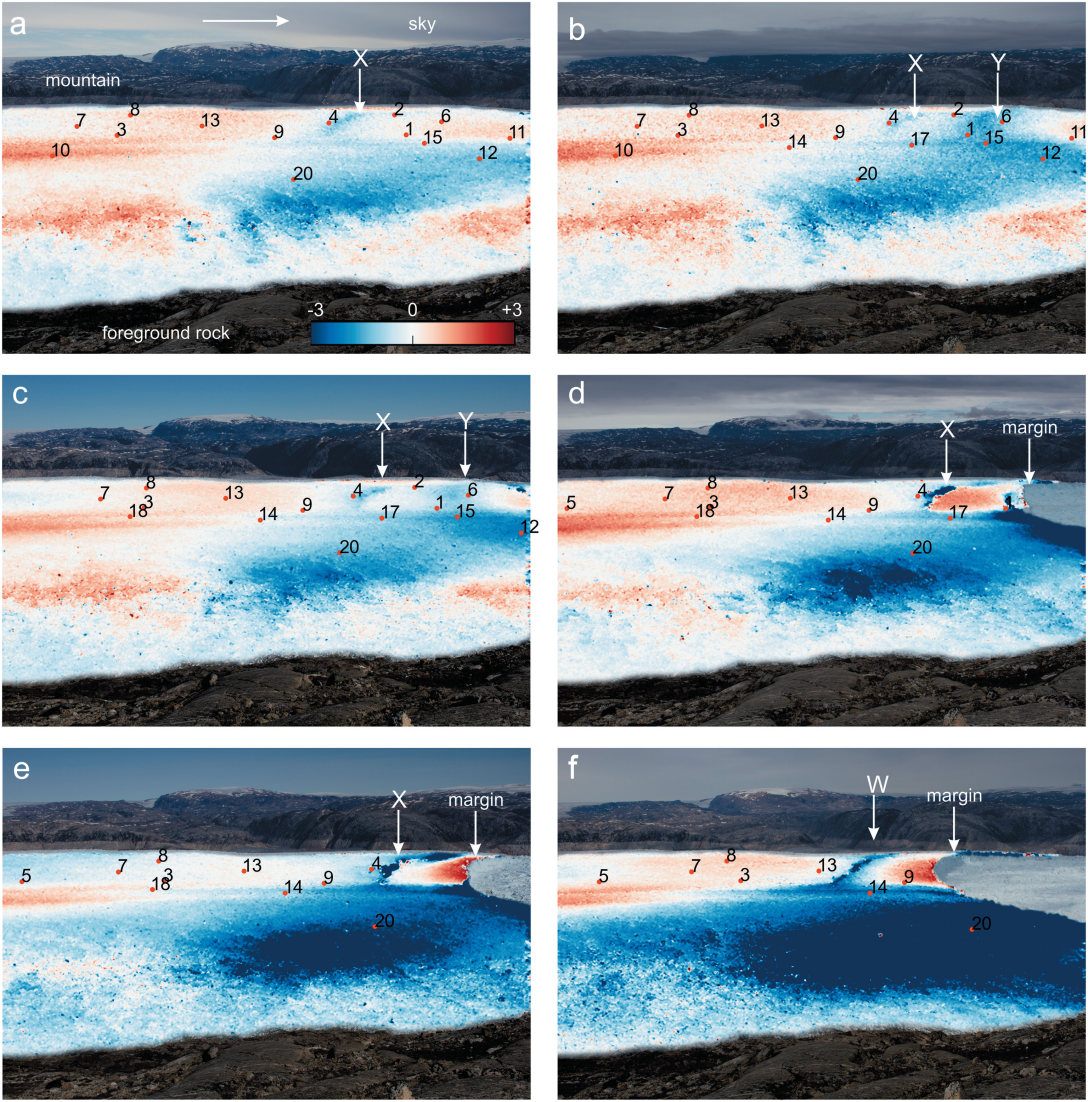
Tracking images showing the vertical component (*y* axis) of flow between images from the sideways looking cameras with node locations superimposed. Only nodes that were transmitting at the time of the images are shown. The images are masked to show tracking results over the glacier surface only to facilitate image interpretation. Background image is second image, and if calving occurs between the two images, masking is done for the smaller extent (second image). Arrow in Figure 7a shows approximate direction of downglacier flow. Scale (in pixels) is the same in all cases. (a) DOY 195–196. Note feature “X” on the northern side of the glacier which shows downward flow. (b) DOY 197–198. Feature “X” has advected downglacier and a new downward moving feature “Y” has formed down glacier of it. Features “X” and “Y” advect downglacier over the intervening period and both continue to show downward movement. (c) DOY 204–205. A series of calving events occur on DOY 205–206 (see Table 1 for a summary of these events). Calving occurs back to feature “Y.” (d) DOY 207–208. Feature “X” continues to advect downglacier. (e) DOY 210–211. Calving events on days 211–212 (Table 1) occur back to feature “X.” A new downward moving feature “W” then forms which advects down glacier. (f) DOY 216–217.

**Figure 8:**
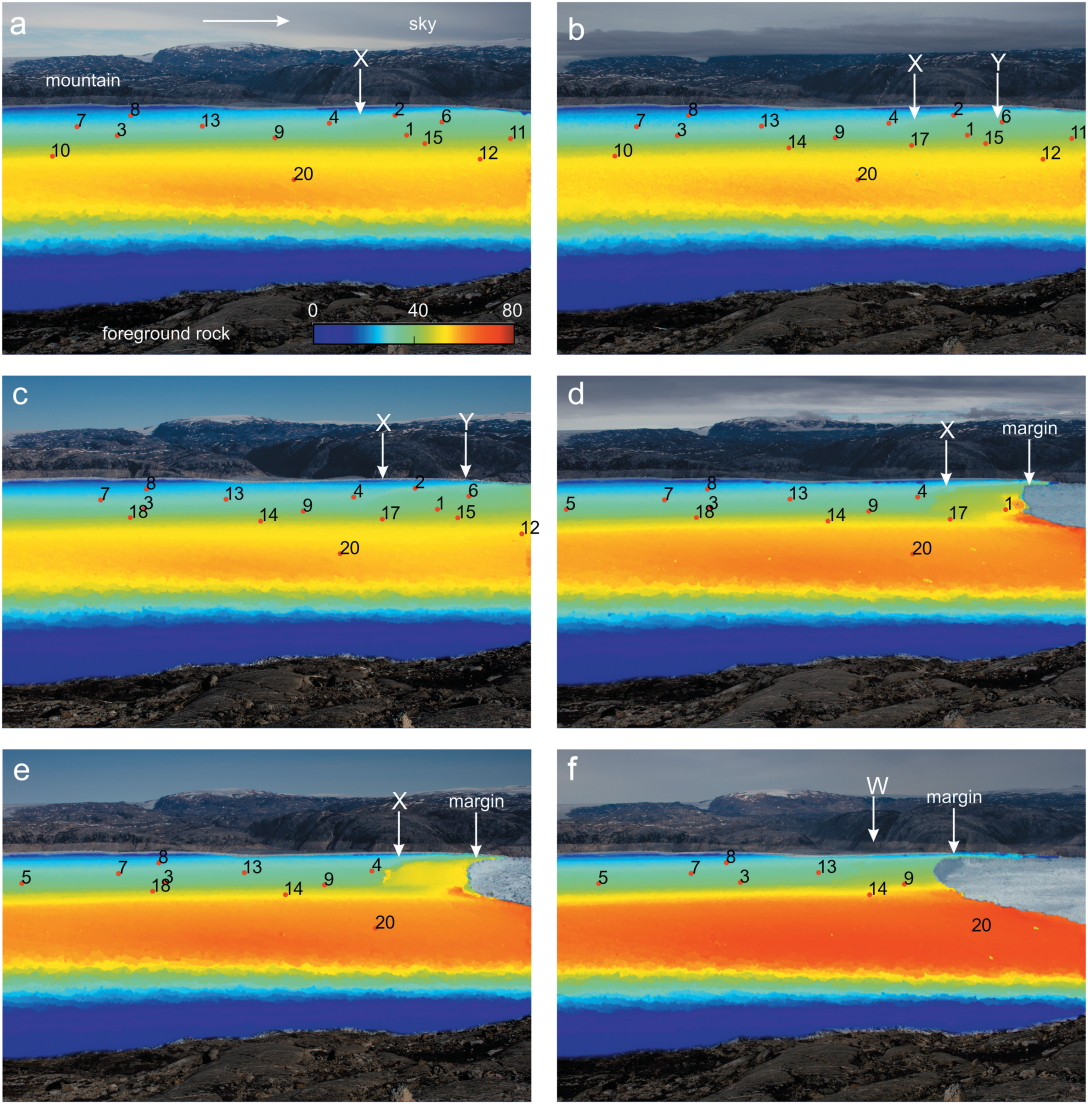
Tracking images showing the horizontal component (*x* axis) of flow between images from the sideways looking cameras with node locations superimposed. Images correspond with those in Figure 7. Scale (in pixels) is the same in all cases. Arrow shows approximate direction of downglacier flow. (a) DOY 195–196. (b) DOY 197–198. (c) DOY 204–205. (d) DOY 207–208. (e) DOY 210–211. (f) DOY 216–217.

**Figure 9:**
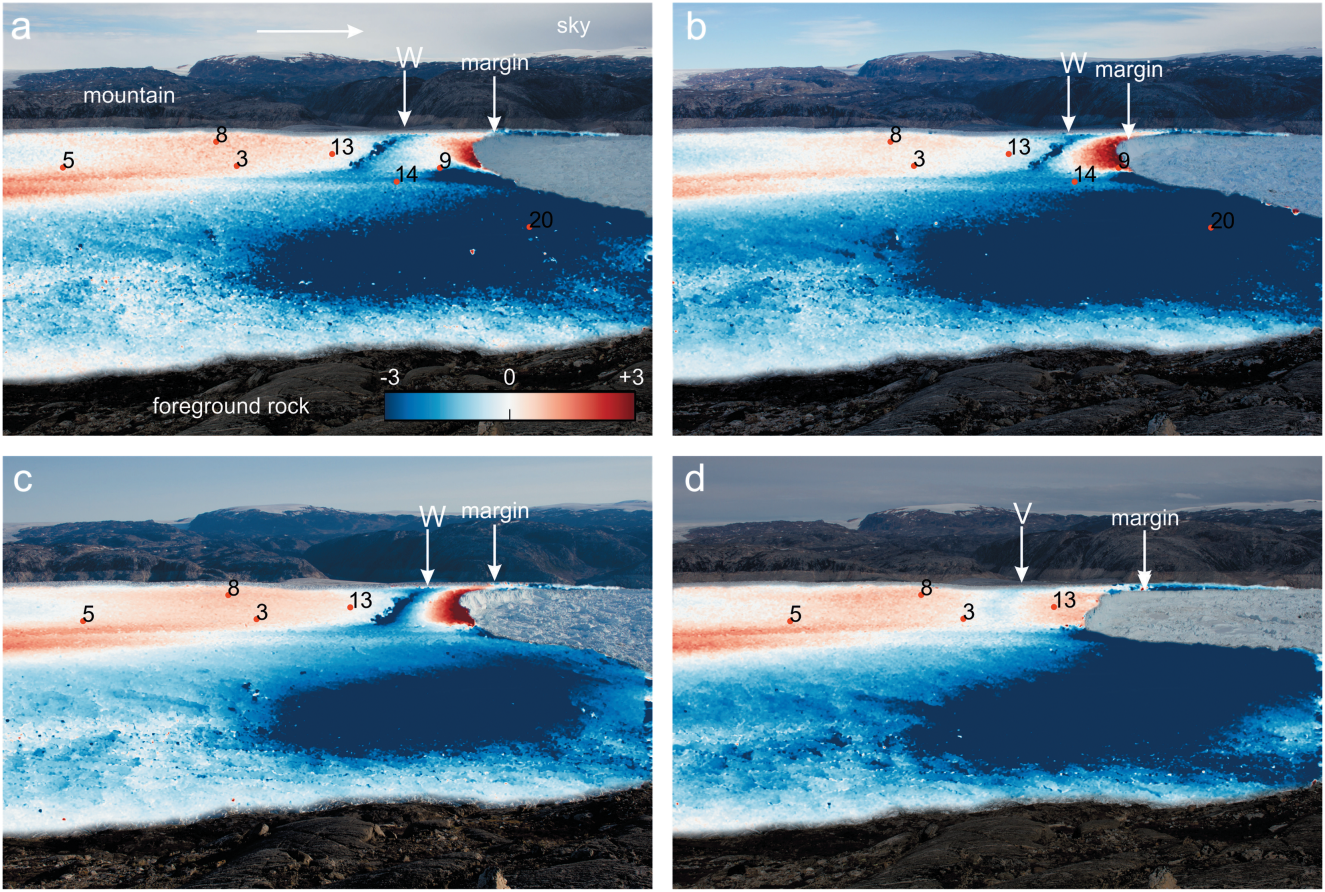
Tracking images showing the vertical component (*y* axis) of flow between images from the sideways looking cameras with node locations superimposed. Only nodes that were transmitting at the time of the images are shown. The images are masked to show tracking results over the glacier surface only to facilitate image interpretation. Background image is second image, and if calving occurs between the two images, masking is done for the smaller extent (second image). Arrow on Figure 9a shows approximate direction of downglacier flow. Scale (in pixels) is the same in all cases. (a) DOY 218–219. Note that the simple pattern of the previous days has been replaced by strong upward movement at the glacier front and neutral movement upglacier of this, and that the feature “W” remains. Calving on DOY 220 occurs back to this intermediate location. (b) DOY 220–221. Feature “W” continues to advect downglacier with strong vertical motion upward at the front and downward at “W.” (c) 223–224. Calving on DOY 226 occurs back to feature “W,” and a new feature “V” forms on DOY 229 and advects downglacier. (d) DOY 231–232.

**Figure 10:**
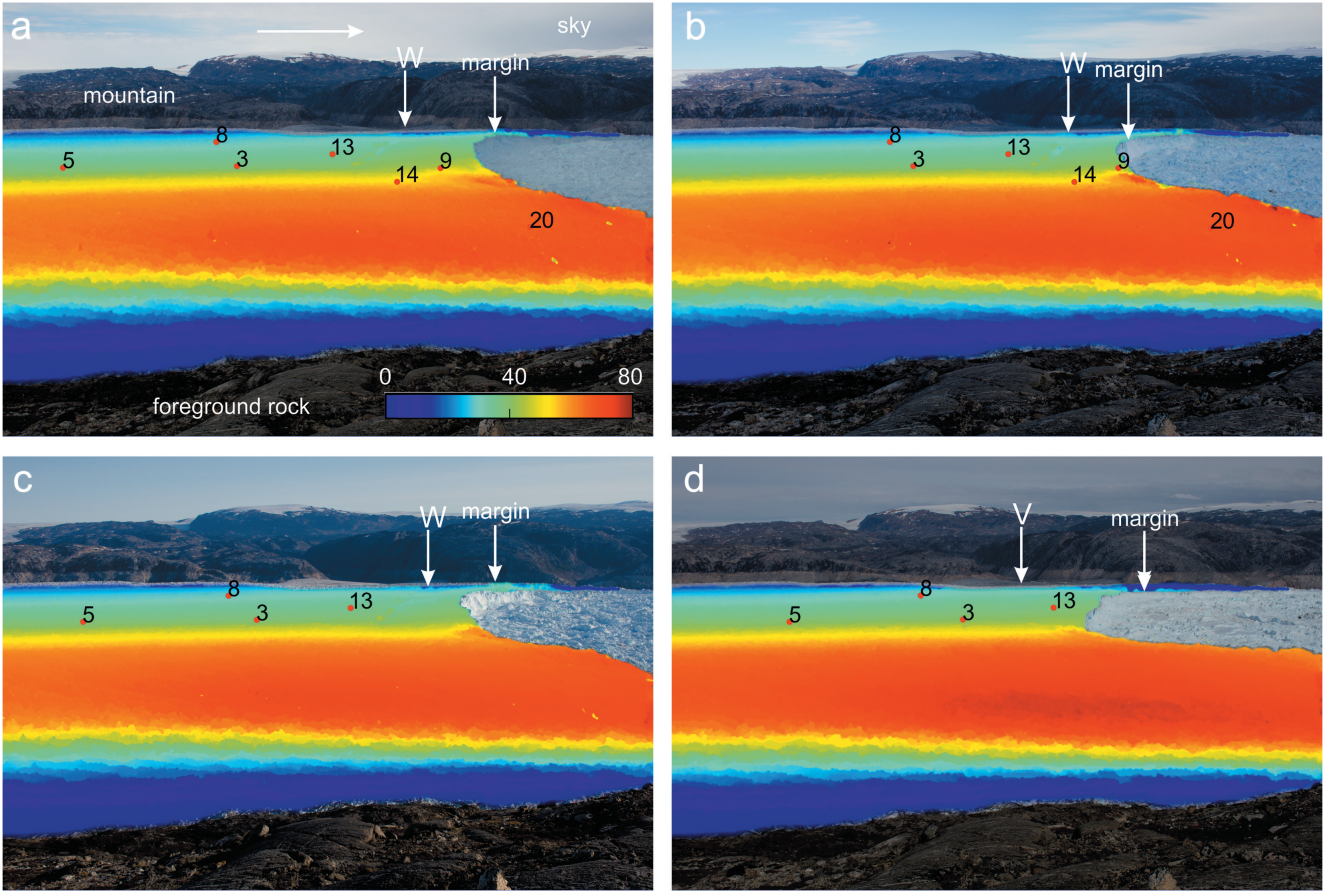
Tracking images showing the horizontal component (*x* axis) of flow between images from the sideways looking cameras with node locations superimposed. Images correspond with those in Figure 9. Scale (in pixels) is the same in all cases. (a) DOY 218–219. (b) DOY 220–221. (c) 223–224. (d) DOY 231–232.

Figures 7, 8, 9, 10 show sample tracking images using one of the sideways‐looking cameras. On the north side of the glacier, where the majority of the GPS network is situated, Figure 7a shows that between DOY 195 and 196 there is a narrow strip of downward movement about 1.4 km from the front that extends across the glacier approximately to the position of the moraine (moraine location marked on Figure 1), with upward flow both upglacier and downglacier of this (feature “X” on Figure 7a). By the next image (DOY 197–198), this region has advected downglacier and a new region of downward flow (feature “Y”) has formed downglacier of the first region about 0.7 km from the glacier front again extending across the north side of the glacier to the moraine (Figure 7b). Nodes 1, 15, and 16 were located in the region where feature “Y” forms. These two features continue to advect downglacier until the tracking images at 11:00 between DOY 204 and 205 (Figure 7c). A series of calving events follow starting on the evening of DOY 205 and continuing into DOY 206 (Table 1). Subsequent images show that calving occurred back to feature “Y” (the downglacier of the two features) and appeared to have been constrained laterally by the location of the moraine (Figure 7d). Downglacier flow rates on the horizontal tracking images show faster flow rates downglacier of feature “X,” which is clear by DOY 207–208 (feature “X”) (Figures 8a–8e). There was significant horizontal speed up across the glacier at this calving event (Figure 8d).

The feature (“X”) continues to advect downglacier with strong uplift downglacier of it and strong downward movement associated with its location (Figure 7e). At the time node 9 was located upglacier of feature “X.” Horizontal flowrates show a jump in flow speed across the feature (~50%) (Figure 8e). By DOY 210 a rift is very clear on photographs of the glacier surface. On DOY 211 and 212, the glacier calves in steps back to the surface feature “X” and on the tracking image between DOY 211 and 212, a new downward moving feature has formed about 1.0 km from the glacier front (feature “W” on Figure 7f) close to node 13. At this time node 9 is downglacier of the feature and on a part of the glacier showing clear uplift. The feature (“W”) again advects downglacier (Figure 9a) with a rift visible on the glacier surface from around DOY 216 and becoming large. Despite its existence, calving on DOY 220 does not occur back to this feature but to an intermediate location. There is some indication of this calving location on the tracking image on DOY 218–219 as a transition from neutral movement to strong upward movement (Figure 9a) approximately at the location of node 9. After this calving event, feature “W” continues to advect downglacier (Figure 9b), with a strengthening signal of vertical motion (Figure 9c) but without a strong horizontal increase in speed across the feature (Figures 10b and 10c), and calving on DOY 226 then occurs back to feature “W.” A new feature (“V”) can be seen from DOY 229–230 which forms between nodes 3 and 13 (Figure 9d) and advects downglacier until the end of the sequence on DOY 245.

## 4 Conceptual Model

**Figure 11:**
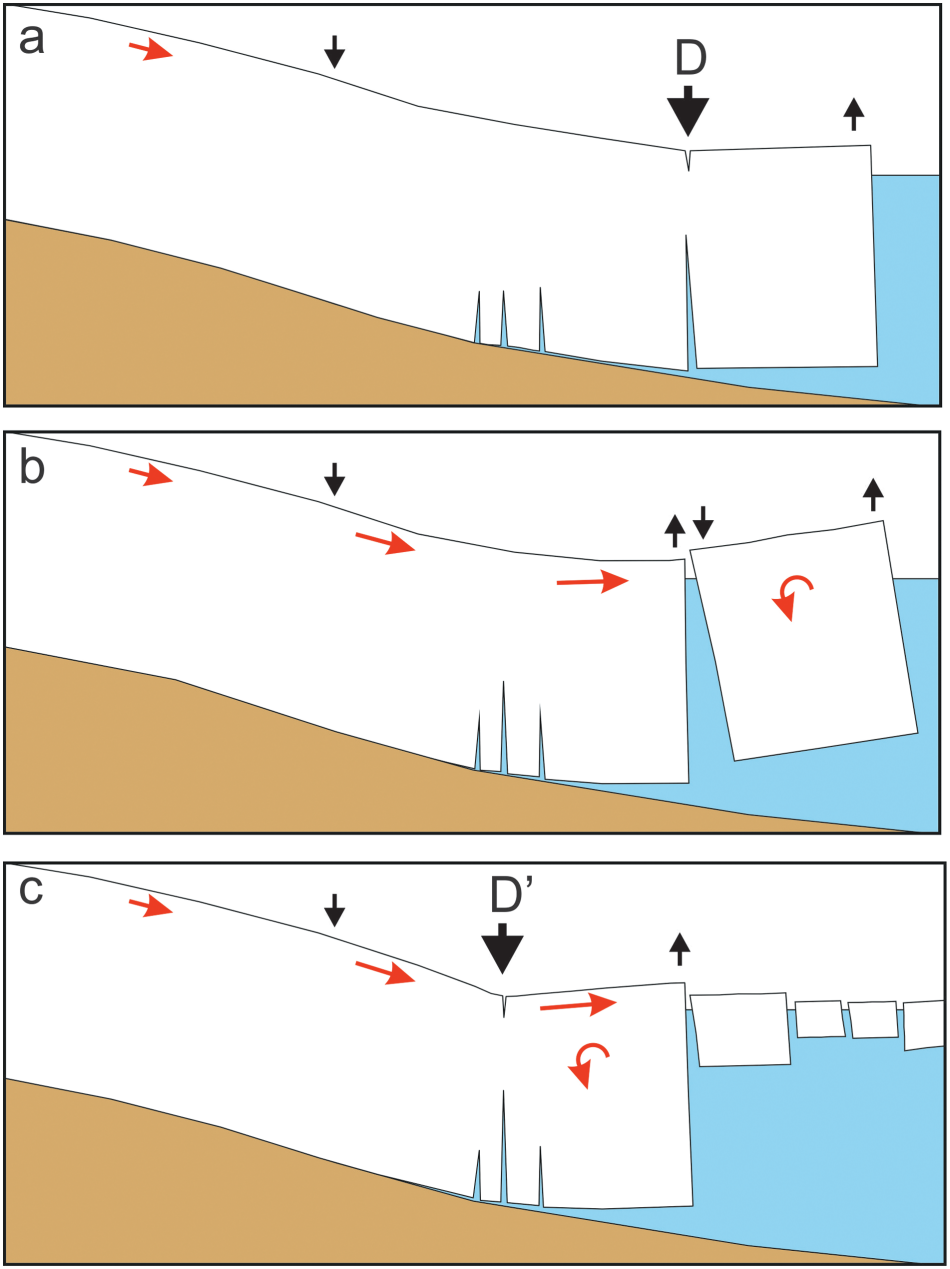
Qualitative model of glacier calving. (a) In the period prior to calving, the front of the glacier uplifts and rotates exploiting a bottom crevasse. The surface expression of the bottom crevasse is a flexion zone (here marked D), a region of the glacier that is lowering and forms a depression, and eventually a rift at the glacier surface. (b) Calving occurs back to this flexion zone D and is normally bottom out. Either coincident with calving or shortly afterward (c) a new region of flexure forms D′ exploiting a bottom crevasse, and the front of the glacier again starts to lift and rotate. The ice mélange in front of the glacier is a mix of sea ice and calved iceberg parts.

We interpret these data within the framework of the model introduced by *James et al*. [2014] for calving driven by buoyant flexure probably associated with the exploitation and propagation of basal crevasses. The model provides a compelling and complete explanation for the data, and we are able to provide not only evidence for its veracity but also further constraints on the process. Figure 11 presents a cartoon summary of this model. Bottom crevasses are expected to form in areas of extending flow near the grounding line [*Van der Veen*, 1998]. We know that the front of Helheim Glacier was not grounded on the north side of the moraine as many nodes show tidal modulation of their vertical position. The model requires that the front of the glacier is out of buoyant equilibrium, and therefore, buoyancy forces tend to cause the front to lift and rotate, a process that exploits bottom crevasses (Figure 11a). At the upper surface of the glacier, a depression forms (we term this a “flexion zone” (indicated as D and D′ on Figure 11) where the surface is lowering. Such zones will advect and develop downglacier with glacier flow often developing into surface rifts [*Joughin et al*., 2008]. Calving occurs back to this location (Figure 11b) and a new flexion zone forms upglacier (Figure 11c). Iceberg calving normally occurs bottom out (Figure 11b) and is associated with the production of a glacial earthquake.

The simplest explanation for the buoyancy forces required is that the rapid flow of the glacier front (25+ m/d) pushes the ice into deeper water at a depth below buoyant equilibrium (as depicted in Figure 11) faster than the glacier can respond by viscous flow. *James et al*. [2014] discuss the bed data in this area of Helheim and conclude that its accuracy is insufficient to ascertain whether the bed is deepening beneath the glacier at this location. However, changes in bed depth in conjunction with bottom melting together with dynamic thinning must be sufficient to allow the front of the glacier to unground as we observe. Note that melting of the upper surface will operate to compound the disequilibrium in buoyancy, whereas basal melting will tend to counteract it.

Thus, our explanation for most periods of time when our GPS nodes are uplifting (e.g., Figures 4a and 5) is that they are situated on regions of the glacier that are downglacier of a flexion zone. Furthermore, each nontidally driven inflexion in the rate our nodes uplift (for example, those indicated by letters on Figures 4 and 5) can be explained in terms of this model. However, for completeness we discuss three other possible scenarios for the front of the glacier uplifting as observed and explain why they do not satisfactorily explain our observations.

1. The front of the glacier uplifts because the ice mélange in front of the glacier provides sufficient backstress to retard glacier flow. Flow retardation would cause compressional thickening. Counterevidence is provided by our *x*‐component tracking images (Figures 8 and 10) which show that in general downglacier flow increases to the glacier front (and into the mélange beyond). Thus, there is no indication of the decreasing flow rates that would of necessity accompany thickening by compression caused by retardation of the glacier front. Note also that the mélange is highly mobile during calving events, which causes the decorrelation of tracking in this region using images immediately before and after calving events.
2. The front of the glacier is advancing onto a bedrock high causing the front to uplift. This cannot be the case. As the front of the glacier retreats through the summer, the location of the region uplifting retreats upglacier, and regions where uplift occurs overlap in space with regions where nodes move downslope at other times in the data series (Figure 2a). Furthermore, it is impossible to reconcile the increasing amplitude of tidal oscillations seen in node 9 (Figure 5a) with the rapidity of grounding that would be required to achieve the 2.0 m/d of uplift seen at the end of the record between DOY 220 and 221.
3. The icebergs are completely detached throughout the sequence, and the uplift seen is caused by a slow rotation of each iceberg as a rigid body [cf. *Bassis and Jacobs*, 2013]. We consider this unlikely for three reasons. First, rotation of an iceberg in this manner would be expected to occur about an axis close to the center of the iceberg meaning that the pattern of upward and downward movement should be approximately equal. We often see a narrow zone of downward movement with the whole of the downglacier part uplifting (see, e.g., Figures 7c–7f). Second, there is usually no visible manifestation of the location of the flexion zone at the surface until sometime after it is visible in tracking images, whereas if the iceberg was completely detached we would expect this to be visible throughout the sequence. Third, it is hard to explain the formation of a new flexion zone downglacier of an already existing one in a location that has shown no previous downward movement if both blocks were already separate (e.g., Figures 7a and 7b).

## 5 Data Interpretation

At the start of our data series the vertical tracking images clearly show a narrow region of downward movement on the north side of the glacier on DOY 195–196 (“X” on Figure 7a). This feature and other similar features seen on the tracking images (Figures 7 and 9) are interpreted as flexion zones (equivalent to D and D′ in Figure 11) probably formed by the exploitation of basal crevasses. Nodes near this location should have experienced downward movement (e.g., on DOY 195–196, node 9, Figure 5a, also node 4), while nodes located downglacier should have experienced upward movement (e.g., on DOY 195–196, node 11, Figure 4b, also node 2) as the front of the glacier lifted. The flexion features seen on tracking images advected downglacier as expected, and eventually, visual surface expression can be seen in the form of rifting and crevassing (Figure 6, especially 6a and 6d–6f); typically, calving then occurred back to this feature. After calving, although not necessarily straight away, a new region of downward movement (a new flexion zone) formed and the process reoccurred (Figure 11).

Twice in our data series something slightly different happened. Rather than the glacier calving back to the downward flexion feature seen in tracking, a new flexion feature formed downglacier and calving initially occurred back to this new feature. The clearest example of this occurred between DOY 196 and 197 (compare Figures 7a and 7b) when flexion feature “Y” formed downglacier of the feature “X” which was apparent at the start of our data series. Both flexion features then persisted and moved downglacier for 7 days, until the next calving event on DOY 205 occurred back to the newer feature, leaving the original feature “X” still advecting downglacier (Figures 7b and 7c). This new feature “Y” was also probably caused by the exploitation of a basal crevasse downglacier of the original flexion zone. The period between DOY 197 and 205 was the only protracted period in our data when we see two locations of downward movement (two flexion zones). All of nodes 1, 15, and 6 were located in the region of the new feature, and all showed synchronous downward inflexion in the rate of change in vertical height on DOY 197.6. Thus, the feature appeared to form rapidly and affected the dynamics of the whole lower part of the glacier.

The second example of a feature forming downglacier of an already existing feature explains the downward inflexion in the vertical position of node 9 on DOY 218 (“C” on Figure 5a). Prior to the formation of the new feature, node 9 was situated in a region of strong upward flow at +0.68 m/d. When the feature formed it manifested in the tracking data as a region of neutral flow (Figure 9c). For 18 h subsequent to it forming, node 9 moved downward at a rate of −0.57 m/d until the glacier calved back to this new position (Figure 5a). Node 9 then remained balanced right at the glacier front (Figure 6f) experiencing upward flow at ~2.0 m/d until it was lost on DOY 221.

Our data are completely explainable using the calving model described in the previous section. As examples we describe the records from nodes 11, 1, and 9 (Figures 4 and 5).

### 5.1 Node 11

Node 11 was the node installed closest to the front of the glacier. On installation it was located downglacier of flexion zone “X” (Figure 7a), and the node experienced uplift for the entirety of the data record (Figure 4a). The formation of flexion zone “Y” did not have any impact on the node's rate of uplift, which was ~0.26 m/d until calving events on DOY 205–206.

### 5.2 Node 1

Node 1 was installed close to the flexion zone “X” (Figure 7a), and the node was downward moving for the entirety of its data record. The node was located in the position that flexion zone “Y” formed in on DOY 197 (Figure 7b), and its formation was temporally coincident with an increase in the node's submergence rate between 197 and 197.6 (it is difficult to give a precise timing for the increase) (“A” on Figure 4b).

### 5.3 Node 9

The node was installed upglacier of flexion zone “X” and of flexion zone “Y” which subsequently formed downglacier (Figures 7a–7c), and it moved downslope throughout the initial 18 days of our data series. Tidal influence on the node became clearer through the record. The calving events on DOY 205–206 were coincident with a long‐term speed up. These calving events removed the frontal portion of the glacier back to zone “Y;” however, zone “X” was still downglacier of node 9 (Figures 7d–7f). The node continued to move downslope, and over the 18 days its vertical speed was −0.25 m/d until calving events (Box B) on DOY 211 and 212 (Figure 5a). These calving events as well as that on DOY 215 caused short periods of increased flow as well as a long‐term speed up. After the events on DOY 211/212 node 9 was situated downglacier of the new flexion zone “W” (Figure 7f) and it began to uplift with a speed of +0.68 m/d (Figure 5a). Inflexion C in the height of Figure 5a has been discussed above and resulted from the formation of a new feature downglacier of “W.” The calving event on DOY 220 left node 9 teetering on the edge of the glacier (Figure 6f); at this time the node was rising at 2.0 m/d (Figures 5a and 10b). The node survived 1.3 days in this precarious position before being lost on DOY 221. Its loss was not due to any detectable calving event. It is possible that crumbling of the front caused it to calve off as we cannot detect very small events. It is equally likely that such a high rate of elevation change may have resulted in slopes sufficient for the node to fall over or slide off the glacier surface. There are no clues in the data series as to why it is lost.

## 6 What Constraints Can Our Data Place on the Conceptual Model?

### 6.1 Geometry and Formation of Flexion Zones

Flexion zones are interpreted as the surface expression of the glacier breaking at depth most likely by the enlargement and exploitation of basal crevasses. They are visible in tracking data by the presence of narrow bands of subsiding ice. Over time visible surface effects develop through the widening of surface crevasses and rifting (Figure 6). Calving usually occurs at the location of flexion zones and is assumed to occur when basal crevasses reach the base of the surface feature. Flexion zones are seen only on the north side of the glacier. Nodes installed south of the moraine (12 and 20) do not display vertical modulation of their position at tidal frequencies, and indeed only very weak horizontal modulation, if any. It is therefore likely that this side of the glacier is grounded. As expected flexion zones seem only to occur where the glacier has an ungrounded front.

Flexion zones do not form at the same location or with the same geometry on each occasion. Furthermore, different flexion zones appear to have different characteristics. At times the flexion zones are narrow (e.g., feature W in Figures 7f and 9a–9c), whereas at other times they are much wider (e.g., feature Y in Figures 7b and 7c). These differences could reflect the nature of the crevassing at the glacier base and its development through the body of the glacier. For example, it is possible the widest flexion zones reflect multiple crevasses propagating at the glacier bed.

Our GPS nodes show by their tidally modulated vertical movement that the glacier can be ungrounded both upglacier and downglacier of the flexion zone as it propagates downglacier, and demonstate unequivocally that it is buoyancy forces that result in the rotation of the calving ice. The formation of flexion zone “Y” on DOY 197 also clearly shows such a zone can form downglacier of the grounding line (Figure 7b). A new flexion zone forms on DOY 211 (Figure 10a), during a period of neap tides when tidal modulation of the glacier is muted (e.g., Figure 5a). Because of this it is not really possible to state whether the grounding line is upglacier of the feature at this time, although several nodes upglacier of it had shown vertical modulation prior to its formation. The final feature to be formed occurs on DOY 229 at a time which is not associated with calving and was also a period of neap tides. Node 13 is located downglacier of the newly formed flexion zone. The node shows weak vertical modulation of ~0.1 m prior to the formation of the zone, whereas after its formation there is a single tidal rise of 1.3 m peak‐to‐peak followed by cycles of ~0.3 m. It would appear that flexion zones form in places where the ice is ungrounded, i.e., downglacier of the location where vertical tidal modulation can be identified, and that their formation immediately allows uplift to occur, presumably partially relieving the disequilibrium in buoyancy forces.

Tracking images show that flexion zones on the surface can widen and the rate of their deepening can increase as they develop toward calving; however, this is not always the case. For example, the flexion zone formed after the calving event on DOY 220 widens and the rate of deepening increases as it propagates downglacier. Other flexion zones widen, but there is a maximum in the rate of deepening, and some flexion zones remain narrow. There can also be a contrast in velocity across the flexion zone; this is the case for flexion zone “X” after DOY 205 (Figures 8d and 8e). In this case the increase in velocity across the zone grows as calving approaches. This is the most striking incidence of such a velocity increase; in most cases the contrast in velocity across the zone is much smaller. A strong velocity contrast across the flexion zone is likely to increase the rate at which crevassing and rifting occurs at the surface.

### 6.2 Cavity Formation Downglacier of the Flexion Zone

Node 9 was situated right at the calving margin of the glacier just prior to its loss (Figure 6f). Its vertical displacement, together with coincident and subsequent tracking images, allows us to place some constraints on the size of the cavity that formed beneath the rotating ice in front of the flexion zone in the run up to the calving event on DOY 226. At the end of the time series for node 9, its elevation is some 2.9 m above its starting elevation and 7.2 m above its minimum elevation. The node's minimum elevation occurred just prior to the calving event on the evening of DOY 211 when the new flexion zone “W” forms upglacier of it (Figure 9a). After node 9 is lost on DOY 221, no further sizeable calving event happened until DOY 226 (Table 1). In the day or so prior to its loss, node 9 was rising at a rate of 2.0 m/d (Figure 5a). The tracking images show the ice at the front continued to lift at a similar or faster rate until calving late on DOY 226 (Figures 9d and 9e), more than 5 days later. Thus, by the time of calving the ice front had lifted by at least 15–20 m above its minimum elevation. The cavity size will clearly depend on the bathymetry of the fjord beneath the glacier as well as the rate and spatial distribution of basal melt. However, most of Sermilik Fjord has low slopes in the downglacier direction [*Schjoeth et al*., 2012], and so it is likely that the cavity underneath the lifting ice also has a minimum size of about 15–20 m at its downglacier end.

## 7 Discussion

### 7.1 Calving Criteria

Various models of glacier calving [*Nick et al*., 2009, 2010; *Otero et al*., 2010; *Cook et al*., 2012, 2014] have used the crevasse‐depth calving criterion introduced by *Benn et al*. [2007]. The model essentially is driven by surface crevasses with calving occurring when the depth of these surface crevasses reaches the waterline. The criterion is sensitively dependent on crevasse water content [e.g., *Cook et al*., 2012]. *Krug et al*. [2014] present a more sophisticated model that combines continuum damage mechanics and fracture mechanics but still represents calving as a process caused by the propagation of surface crevasses. Our data, together those presented by *James et al*. [2014], bring into question the validity of this calving criterion at Helheim Glacier and suggest a calving criterion that includes basal crevassing is required. *Nick et al*. [2010] used such a criterion stipulating that calving occurred when basal and surface crevasses penetrated the full thickness of the glacier, a criterion that would be supported by our observations. The *Nick et al*. [2010] model represents a field of surface and basal crevasses, which allows the simplifying assumption that that crevasses propagate until the net longitudinal stress is zero [*Benn et al*., 2007]. Basal crevasses will form close to the glacier's grounding line and where ice extension rates are high [*Van der Veen*, 1998], so that we expect them in the area we have instrumented at Helheim Glacier. Adapting the *Nick et al*. [2010] model, the penetration height of basal crevasses, *d*, will be

(1) $$d=\frac{\rho_{i}}{\rho_{w}-\rho_{i}}\left(\frac{R_{xx}}{\rho_{i}g}-H_{\text{ab}}\right)$$

where *ρ*_*i*_. and *ρ*_*w*_. are the density of glacier ice and sea water, respectively, *g* is gravitational acceleration, *R*_*xx*_. is the resistive stss, which is related to the longitudinal stretching rate, and *H*_*ab*_ is the height above buoyancy is defined as

(2) $$H_{\text{ab}}=H-\frac{\rho_{w}}{\rho_{i}}D$$

where *H* is the glacier thickness and *D* is the depth of the glacier ice in the water. Note that if the ice is out of buoyant equilibrium with *D* deeper than expected, as our results suggest, *H*_*ab*_ is negative. Provided formation of basal crevasses occurs before substantial flexure of the glacier takes place, these basal crevasses will penetrate further up into the ice than if the ice were floating at equilibrium or grounded. Significant flexure during crevasse formation would require a more sophisticated treatment which is beyond the scope of this paper.

For water density of 1028 kg/m^3^ and ice density of 920 kg/m^3^, if the glacier is 5 m out of buoyant equilibrium, which our data suggest would not be unreasonable, these simplified equations suggest that crevasses would penetrate an additional ~43 m into the ice body. Furthermore, the extra penetration of surface crevasses is only 1.1 m for every 1 m of water in the surface crevasse, whereas for every meter the base of the glacier is below buoyant equilibrium, the crevasses penetrate an extra 8.5 m, suggesting that if the glacier is driven deeper than buoyant equilibrium there is a strong likelihood of basal crevasses dominating the calving mechanism. It also suggests that flexion zones are likely to form in locations where the ice is ungrounded and where its base is at a maximum depth below flotation. Note that calving by this process will tend to keep the glacier front margin close to but downglacier of the grounding line of the glacier.

Note that the likelihood of ice being driven to depths below flotation and calving in the manner described will depend on a number of factors including the flow speed of glacier, the rate of dynamic thinning, the rate at which the ice can respond by creep, and the rates of surface and basal melt, as well as the bathymetry of the fjord. As buoyancy forces build up, the glacier can respond to these either slowly by creep or by the brittle processes we have observed [*James et al*., 2014]. Where creep flow is sufficient to dissipate the buoyancy forces, the glacier may tend to form a floating tongue.

The buoyancy‐force‐induced calving we observe is associated with the production of glacial earthquakes. Thus, it would appear that this calving mechanism is becoming more prevalent in Greenland, as the number of such glacial earthquakes is increasing both spatially and temporally as tidewater glaciers retreat back to their grounding lines [*Ekström et al*., 2006; *Veitch and Nettles*, 2012].

### 7.2 Mixing and Melting in the Basal Cavity

Water within Sermilik Fjord is three layered, with water deeper than ~250 m being warm water of subtropical origin [*Straneo et al*., 2010]. Typical potential temperatures of this layer are 3.5–4.0 °C where they have been measured [*Straneo et al*., 2010, 2011]. Measurements have been taken along transects in Sermilik Fjord within ~50 km of the glacier front, as well as a few opportunistic measurements within Helheim Fjord itself up to ~10 km from the glacier front [*Straneo et al*., 2011]. Thus, assuming that this stratification persists to the glacier front, the opening of the basal cavity will tend to bathe the base of the glacier in warm, salty water and may increase the opening rate of basal crevasses.

## 8 Conclusions

Iceberg calving at Helheim Glacier during summer 2013 was consistent with a mechanism dominated by buoyant flexure, which causes the upward rotation of ungrounded ice downglacier of flexion zones. These flexion zones seem to be formed by the exploitation of basal crevasses and are characterized at the surface by downward displacement of the ice through a combination of longitudinal stretching and surface rifting. These basal crevasses propagate until they meet the surface crevasse / rift and calving then occurs, usually back to the flexion zone.

This model provides a consistent and complete explanation for data from GPS and oblique cameras collected at the margin of Helheim Glacier during summer 2013. The flexion zones are clearly apparent by tracking in the vertical image direction using the sideways‐looking camera images. GPS nodes were located both downglacier and upglacier of the flexion zones. In general, nodes downglacier of the dominant flexion zone uplift and those upglacier of it experience downward movement. After a calving event a new flexion zone is formed and node can switch rapidly from uplift to downward movement when this new zone forms upglacier of the node's location. The opposite switch (from uplift to downward movement) or an increase in the rate of downward movement can also occur if a new flexion zone is formed downglacier of the node.

The data allow the identification and characterization of the flexion zones and quantification of the rates of vertical displacement that result from their formation and subsequent calving. We suggest that this process of calving by buoyant flexure and the exploitation of basal crevasses will dominate whenever ice is driven below buoyant equilibrium by flow into deep water, by dynamic thinning, or by surface melting. The process is of increasing importance in Greenland as tidewater glaciers retreat back to their grounding lines. Calving by this process will tend to keep the glacier front margin close to the grounding line of the glacier.

## References

[jgrf20394-bib-0002] Andersen, M. L. , T. B. Larsen , M. Nettles , P. Elosegui , D. van As , G. S. Hamilton , L. A. Stearns , J. L. Davis , A. P. Ahlstrom , and J. de Juan (2010), Spatial and temporal melt variability at Helheim Glacier, East Greenland, and its effect on ice dynamics, J. Geophys. Res., 115, F04041, doi:10.1029/2010JF001760.

[jgrf20394-bib-0003] Baronti, P. , P. Pillai , V. W. C. Chook , S. Chessa , A. Gotta , and Y. Fun Hu (2007), Wireless sensor networks: A survey on the state of the art and the 802.15.4 and ZigBee standards, Comput. Commun., 30, 1655–1695.

[jgrf20394-bib-0004] Bar‐Sever, Y. E. , P. M. Kroger , and J. A. Borjesson (1998), Estimating horizontal gradients of tropospheric path delay with a single GPS receiver, J. Geophys. Res., 103(B3), 5019–5035, doi:10.1029/97JB03534.

[jgrf20394-bib-0005] Bassis, J. N. , and S. Jacobs (2013), Diverse calving patterns linked to glacier geometry, Nat. Geosci., 6, 833–836, doi:10.1038/NGEO1887.

[jgrf20394-bib-0006] Benn, D. I. , N. R. J. Hulton , and R. H. Mottram (2007), “Calving laws”, “sliding laws” and the stability of tidewater glaciers, Ann. Glaciol., 46, 123–130.

[jgrf20394-bib-0007] Bertiger, W. , S. Desai , B. Haines , N. Harvey , A. Moore , S. Owen , and J. Weiss (2010), Single receiver phase ambiguity resolution with GPS data, J. Geodes., 84(5), 327–337, doi:10.1007/s00190-010-0371-9.

[jgrf20394-bib-0053] Bevan, S. L. , A. J. Luckman , and T. Murray (2012), Glacier dynamics over the last quarter of a century at Helheim, Kangerdlugssuaq and 14 other major Greenland outlet glaciers, Cryosphere, 6, 923–937, doi:10.5194/tc-6-923-2012.

[jgrf20394-bib-0009] Bevan, S. L. , A. J. Luckman , S. A. Khan , and T. Murray (2015), Seasonal dynamic thinning at Helheim Glacier, Earth Planet. Sci. Lett., 415, 47–53, doi:10.1016/j.epsl.2015.01.031.

[jgrf20394-bib-0010] Boyce, E. S. , R. J. Motyka , and M. Truffer (2007), Flotation ad retreat of a lake‐calving terminus, Mendenhall Glacier, southeast Alaska U.S.A., J. Glaciol., 53(181), 211–224.

[jgrf20394-bib-0011] Chen, G. (1998), GPS kinematic positioning for the airborne laser altimetry at Long Valley, California, PhD thesis, Mass. Inst. of Technol., Cambridge.

[jgrf20394-bib-0012] Cook, S. , T. Zwinger , I. C. Rutt , S. O'Neel , and T. Murray (2012), Testing the effect of water in crevasses on a physically based calving model, Ann. Glaciol., 53, 90–96.

[jgrf20394-bib-0013] Cook, S. , I. C. Rutt , T. Murray , A. Luckman , T. Zwinger , N. Selmes , A. Goldsack , and T. D. James (2014), Modelling environmental influences on calving at Helheim Glacier in eastern Greenland, Cryosphere, 8, 827–841.

[jgrf20394-bib-0014] Dach, R. , E. Brockman , S. Schaer , G. Beutler , M. Meindl , L. Prange , H. Bock , A. Jäggi , and L. Ostini (2009), GNSS processing at CODE: Status report, J. Geod., 83, 353–365, doi:10.1007/s00190-008-0281-2.

[jgrf20394-bib-0015] de Juan, J. , et al. (2010), Sudden increase in tidal response linked to calving and acceleration at a large Greenland outlet glacier, Geophys. Res. Lett., 37, L12501, doi:10.1029/2010GL043289.

[jgrf20394-bib-0016] Ekström, G. , M. Nettles , and V. C. Tsai (2006), Seasonality and increasing frequency of Greenland glacial earthquakes, Science, 311(5768), 1756–1758. 16556839 10.1126/science.1122112

[jgrf20394-bib-0017] Farneback, G. (2003), Two‐frame motion estimation based on polynomial expansion, *Proceedings of the 13th Scandinavian Conference on Image Analysis*, Gothenburg, Sweden, 363–370.

[jgrf20394-bib-0018] Howat, I. M. , I. Joughin , S. Tulaczyk , and S. Gogineni (2005), Rapid retreat and acceleration of Helheim Glacier, east Greenland, Geophys. Res. Lett., 32, L22502, doi:10.1029/2005GL024737.

[jgrf20394-bib-0019] Howat, I. M. , I. R. Joughin , and T. A. Scambos (2007), Rapid changes in ice discharge from Greenland outlet glaciers, Science, 315, 1559–1561. 17289940 10.1126/science.1138478

[jgrf20394-bib-0020] Jackson, R. , F. Straneo , and D. A. Sutherland (2014), Externally forced fluctuations in ocean temperature at Greenland glaciers in non‐summer months, Nat. Geosci., 7(7), 503–508, doi:10.1038/NGEO2186.

[jgrf20394-bib-0021] James, T. D. , T. Murray , N. Selmes , K. Scharrer , and M. E. O'Leary (2014), Buoyant flexure and basal crevassing in dynamic mass loss at Helheim Glacier, Nat. Geosci., 7, 593–596, doi:10.1038/ngeo2204.

[jgrf20394-bib-0022] Joughin, I. , I. Howat , R. B. Alley , G. Ekström , M. Fahnestock , T. Moon , M. Nettles , M. Truffer , and V. C. Tsai (2008), Ice‐front variation and tidewater behavior on Helheim and Kangerdlugssuaq Glaciers, Greenland, J. Geophys. Res., 113, F01004, doi:10.1029/2007JF000837.

[jgrf20394-bib-0023] Krug, J. , J. Weiss , O. Gagliardini , and G. Durand (2014), Combining damage and fracture mechanics to model calving, Cryosphere, 8(6), 2101–2117, doi:10.5194/tc-8-2101-2014.

[jgrf20394-bib-0024] Luckman, A. , T. Murray , R. de Lange , and E. Hanna (2006), Rapid and synchronous ice‐dynamic changes in East Greenland, Geophys. Res. Lett., 33, L03503, doi:10.1059/2005GL025048.

[jgrf20394-bib-0025] Lyard, F. , F. Lefevre , T. Letellier , and O. Francis (2006), Modelling the global ocean tides: Modern insights from FES2004, Ocean Dyn., 56, 394–415, doi:10.1007/s10236-006-0086-x.

[jgrf20394-bib-0026] Martin, I. , T. O'Farrell , R. Aspey , S. J. Edwards , T. D. James , P. Loskot , T. Murray , I. C. Rutt , N. Selmes , and T. Baugé (2014), High‐resolution sensor network for monitoring glacier dynamics, IEEE Sensor. J., 14, 3926–3931, doi:10.1109/JSEN.2014.2348534.

[jgrf20394-bib-0027] Motyka, R. J. , L. Hunter , K. A. Echelmeyer , and C. Connor (2003), Submarine melting at the terminus of a temperate tidewater glacier, LeConte Glacier, Alaska, U.S.A., Ann. Glaciol., 36(1), 57–65.

[jgrf20394-bib-0028] Murray, T. , et al. (2010), Ocean‐regulation hypothesis for glacier dynamics in south‐east Greenland and implications for ice‐sheet mass changes, J. Geophys. Res., 115, F03026, doi:10.1029/2009JF001522.

[jgrf20394-bib-0029] Nettles, M. , and G. Ekström (2010), Glacial earthquakes in Greenland and Antarctica, Annu. Rev. Earth Planet. Sci., 38, 467–491, doi:10.1146/annurev-earth-040809-152414.

[jgrf20394-bib-0030] Nettles, M. , et al. (2008), Step‐wise changes in glacier flow speed coincide with calving and glacial earthquakes at Helheim Glacier, Greenland, Geophys. Res. Lett., 35, L24503, doi:10.1029/2008GL036127.

[jgrf20394-bib-0033] Nick, F. , A. Vieli , I. M. Howat , and I. Joughin (2009), Large‐scale changes in Greenland outlet glacier dynamics triggered at the terminus, Nat. Geosci., 2(2), 110–114, doi:10.1038/NGEO394.

[jgrf20394-bib-0031] Nick, F. M. , C. J. Van der Veen , A. Vieli , and D. I. Benn (2010), A physically based calving model applied to marine outlet glaciers and implications for the glacier dynamics, J. Glaciol., 56(199), 781–794.

[jgrf20394-bib-0032] Nick, F. M. , A. Vieli , M. L. Andersen , I. Joughin , A. Payne , T. L. Edwards , F. Pattyn , and R. S. W. van der Wal (2013), Future sea‐level rise from Greenland's main outlet glaciers in a warming climate, Nature, 497, 235–238, doi:10.1038/nature12068. 23657350 10.1038/nature12068

[jgrf20394-bib-0034] Niell, A. E. (2000), Improved atmospheric mapping functions for VLBI and GPS, Earth Planets Space, 52, 699–702.

[jgrf20394-bib-0035] O'Leary, M. , and P. Christoffersen (2013), Calving on tidewater glaciers amplified by submarine frontal melting, Cryosphere, 7(1), 119–128, doi:10.5194/tc-7-119-2013.

[jgrf20394-bib-0036] Otero, J. , F. J. Navarro , C. Martin , M. L. Cuadrado , and M. I. Corcuera (2010), A three‐dimensional calving model: Numerical experiments on Johnsons Glacier, Livingston Island, Antarctica, J. Glaciol., 56(196), 200–214.

[jgrf20394-bib-0037] Pelto, M. S. , and C. R. Warren (1991), Relationship between tidewater glacier calving velocity and water depth at the calving front, Ann. Glaciol., 15, 115–118.

[jgrf20394-bib-0038] Pritchard, H. D. , R. J. Arthern , D. G. Vaughan , and L. A. Edwards (2009), Extensive dynamic thinning on the margins of the Greenland and Antarctic Ice Sheets, Nature, 461(7266), 971–975, doi:10.1038/nature08471. 19776741 10.1038/nature08471

[jgrf20394-bib-0039] Rignot, E. , and P. Kanagaratnam (2006), Changes in the velocity structure of the Greenland Ice Sheet, Science, 311(5763), 986–990. 16484490 10.1126/science.1121381

[jgrf20394-bib-0040] Rosenau, R. , E. Schwalbe , H.‐G. Maas , M. Baessler , and R. Dietrich (2013), Grounding line migration and high‐resolution calving dynamics of Jakobshavn Isbræ, West Greenland, J. Geophys. Res. Earth Surf., 118, 382–395, doi:10.1029/2012JF002515.

[jgrf20394-bib-0041] Schjoeth, F. , C. S. Andresen , F. Straneo , T. Murray , K. Scharrer , and A. Korablev (2012), Collaborative mapping of the bathymetry of a major Greenland Fjord, Eos Trans. AGU, 93(14), 141–142.

[jgrf20394-bib-0042] Sole, A. , T. Payne , J. Bamber , P. Nienow , and W. Krabil (2008), Testing hypotheses of the cause of peripheral thinning of the Greenland Ice Sheet: Island‐terminating ice thinning at anomalously high rates?, Cryosphere, 2(2), 205–218.

[jgrf20394-bib-0043] Stearns, L. A. , and G. S. Hamilton (2007), Rapid volume loss from two East Greenland outlet glaciers quantified using repeat stereo satellite imagery, Geophys. Res. Lett., 34, L05503, doi:10.1029/2006GL028982.

[jgrf20394-bib-0044] Straneo, F. , G. S. Hamilton , D. A. Sutherland , L. A. Sterns , F. Davidson , M. O. Hammill , G. B. Stenson , and A. Rosling‐Asvid (2010), Rapid circulation of warm subtropical waters in a major glacial fjord in East Greenland, Nat. Geosci., 3, 182–186, doi:10.1038/NGEO764.

[jgrf20394-bib-0045] Straneo, F. , R. G. Curry , D. A. Sutherland , G. S. Hamilton , C. Cenedese , K. Våge , and K. L. Stearns (2011), Impact of fjord dynamics and glacial runoff on the circulation near Helheim Glacier, Nat. Geosci., 4, 322–327, doi:10.1038/NGEO1109.

[jgrf20394-bib-0046] Sutherland, D. A. , and F. Straneo (2012), Estimating ocean heat transports and submarine melt rates in Sermilik Fjord, Greenland, using lowered acoustic Doppler current profiler (LADCP) velocity profiles, Ann. Glaciol., 53(60), 50–58, doi:10.3189/2012AoG60A050.

[jgrf20394-bib-0047] Van der Veen, C. J. (1996), Tidewater calving, J. Glaciol., 42(141), 375–385.

[jgrf20394-bib-0048] Van der Veen, C. J. (1998), Fracture mechanics approach to penetration of bottom crevasses on glaciers, Cold Reg. Sci. Technol., 27(3), 213–223.

[jgrf20394-bib-0054] Veitch, S. A. , and M. Nettles (2012), Spatial and temporal variations in Greenland glacial‐earthquake activity, 1993–2010, J. Geophys. Res., 117, F04007, doi:10.1029/2012JF002412.

[jgrf20394-bib-0049] Velicogna, I. (2009), Increasing rates of ice mass loss from the Greenland and Antarctic Ice Sheets revealed by GRACE, Geophys. Res. Lett., 36, L19503, doi:10.1029/2009GL040222.

[jgrf20394-bib-0050] Warren, C. , D. Benn , V. Winchester , and S. Harrison (2001), Buoyancy‐driven lacustrine calving, Glaciar Nef, Chilean Patagonia, J. Glaciol., 47(156), 135–146, doi:10.3189/172756501781832403.

[jgrf20394-bib-0051] Wolf, P. R. , and B. A. Dewitt (2000), Elements of Photogrammetry, With Applications in GIS, 3rd ed., 608 pp., McGraw‐Hill, New York.

[jgrf20394-bib-0052] Zumberge, J. F. , M. B. Heflin , D. C. Jefferson , M. M. Watkins , and F. H. Webb (1997), Precise point positioning for the efficient and robust analysis of GPS data from large networks, J. Geophys. Res., 102(B3), 5005–5017, doi:10.1029/96JB03860.

